# Combination therapy with normobaric oxygen (NBO) plus thrombolysis in experimental ischemic stroke

**DOI:** 10.1186/1471-2202-10-79

**Published:** 2009-07-15

**Authors:** Norio Fujiwara, Yoshihiro Murata, Ken Arai, Yasuhiro Egi, Jie Lu, Ona Wu, Aneesh B Singhal, Eng H Lo

**Affiliations:** 1 Neuroprotection Research Laboratory, Departments of Neurology, Massachusetts General Hospital, Harvard Medical School, Boston, USA; 2 Neuroprotection Research Laboratory, Departments of Radiology, Massachusetts General Hospital, Harvard Medical School, Boston, USA

## Abstract

**Background:**

The widespread use of tissue plasminogen activator (tPA), the only FDA-approved acute stroke treatment, remains limited by its narrow therapeutic time window and related risks of brain hemorrhage. Normobaric oxygen therapy (NBO) may be a useful physiological strategy that slows down the process of cerebral infarction, thus potentially allowing for delayed or more effective thrombolysis. In this study we investigated the effects of NBO started simultaneously with intravenous tPA, in spontaneously hypertensive rats subjected to embolic middle cerebral artery (MCA) stroke. After homologous clot injection, animals were randomized into different treatment groups: saline injected at 1 hour; tPA at 1 hour; saline at 1 hour plus NBO; tPA at 1 hour plus NBO. NBO was maintained for 3 hours. Infarct volume, brain swelling and hemorrhagic transformation were quantified at 24 hours. Outcome assessments were blinded to therapy.

**Results:**

Upon clot injection, cerebral perfusion in the MCA territory dropped below 20% of pre-ischemic baselines. Both tPA-treated groups showed effective thrombolysis (perfusion restored to nearly 100%) and smaller infarct volumes (379 ± 57 mm^3 ^saline controls; 309 ± 58 mm^3 ^NBO; 201 ± 78 mm^3 ^tPA; 138 ± 30 mm^3 ^tPA plus NBO), showing that tPA-induced reperfusion salvages ischemic tissue and that NBO does not significantly alter this neuroprotective effect. NBO had no significant effect on hemorrhagic conversion, brain swelling, or mortality.

**Conclusion:**

NBO can be safely co-administered with tPA. The efficacy of tPA thrombolysis is not affected and there is no induction of brain hemorrhage or edema. These experimental results require clinical confirmation.

## Background

Intravenous tissue plasminogen activator (tPA) remains the only acute stroke therapy that is approved by the FDA and established to improve clinical outcome [1,2]. However, the use of this thrombolytic agent has been limited by the need to deliver treatment within a narrow therapeutic time window, presently 3 hours, and the excess risk of brain hemorrhage and edema ('reperfusion injury') if treatment is started at delayed time points after stroke [1,2]. It is important to develop strategies that can safely extend the therapeutic time window for tPA and thereby increase the utilization of this effective treatment.

A key factor that increases the risks for brain edema and hemorrhage, and reduces the efficacy of tPA, is the development of substantial cellular necrosis prior to treatment. Preventing early cell death may allow relatively delayed thrombolysis with tPA, without compromising safety. Several animal [3-16] and human [17-19] studies have documented that normobaric oxygen therapy (NBO) therapy is neuroprotective in acute ischemic stroke. Imaging studies indicate that NBO slows down and transiently arrests the process of ischemic cell death[6,14,18,19]. Since it is simple to administer, noninvasive, inexpensive, widely available, and can be started in the field within minutes after stroke symptom onset, NBO is being viewed as a potentially useful and feasible physiological strategy to extend the time window for stroke thrombolysis [20-23].

In this study, we tested the hypothesis that NBO therapy can be safely combined with tPA in an experimental clot-based stroke model in rats. In order for combination NBO plus tPA clinical trials to be initiated, one must first demonstrate that NBO does not interfere with the efficacy or compromise the safety of tPA thrombolysis. The experimental design and results of this study are relevant to the clinical scenario where tPA and NBO are started simultaneously upon hospital arrival.

## Results

**Figure 1:**
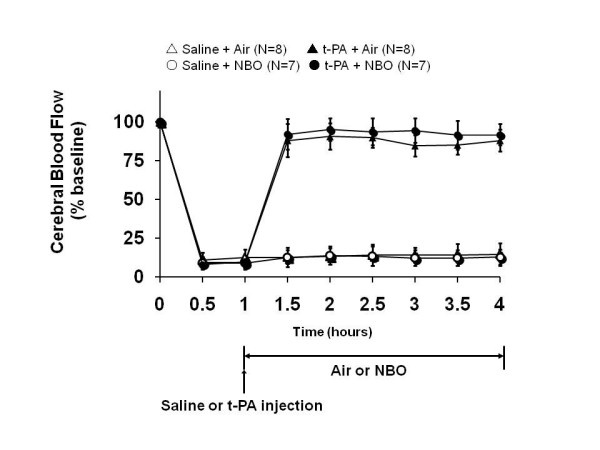
**Cerebral perfusion during embolic focal ischemia and reperfusion after tPA thrombolysis**. Cerebral perfusion as measured with laser Doppler flowmetry (mean ± SD) dropped rapidly below 20% after clot injection. Intravenous tPA therapy almost fully restored perfusion. Saline did not have detectable effects on cerebral perfusion values.

Physiological parameters remained within normal range in all groups (Table 1). Before NBO, arterial pO2 levels were approximately 120 mm Hg in all rats. As expected, NBO rapidly and markedly elevated arterial pO2 levels to above 400 mmHg (Table 1). Upon clot injection, cerebral perfusion in the MCA territory dropped below 20% of pre-ischemic baselines (Figure 1). Cerebral ischemia was stable throughout the 3 hour duration of the study in all animals (note that any animals showing spontaneous recanalization were excluded; see Methods). tPA treatment restored perfusion to almost 100%, but saline treatment did not have detectable effects on cerebral perfusion values (Figure 1). The addition of NBO did not affect the rate or the time course of tPA-mediated reperfusion (Figure 1).

**Table 1:** Physiological Variables

	Group			
	
	Saline + Air	Saline + NBO	t-PA + Air	t-PA + NBO
Body Weight (g)	302.9 ± 8.1	304.0 ± 7.8	303.4 ± 6.6	306.3 ± 5.3

Rectal Temperture (°C)				
before Air/NBO	37.0 ± 0.1	37.0 ± 0.1	37.0 ± 0.1	37.0 ± 0.1
30 min after Air/NBO	37.0 ± 0.1	37.0 ± 0.1	37.0 ± 0.1	37.0 ± 0.1
3 hr after Air/NBO	37.0 ± 0.1	37.0 ± 0.1	37.0 ± 0.1	37.0 ± 0.1

MABP (mmHg)				
Before Air/NBO	182.9 ± 5.6	185.6 ± 5.6	185.9 ± 6.9	183.0 ± 5.1
30 min after Air/NBO	182.5 ± 7.1	187.4 ± 8.0	185.4 ± 8.1	185.6 ± 5.4
3 hr after Air/NBO	183.0 ± 6.3	186.1 ± 7.1	184.5 ± 6.3	182.1 ± 7.8

pH				
Before Air/NBO	7.38 ± 0.01	7.37 ± 0.01	7.38 ± 0.01	7.38 ± 0.01
30 min after Air/NBO	7.38 ± 0.01	7.37 ± 0.01	7.38 ± 0.01	7.38 ± 0.01
3 hr after Air/NBO	7.38 ± 0.01	7.37 ± 0.01	7.37 ± 0.01	7.38 ± 0.01

pO2 (mmHg)				
Before Air/NBO	121.8 ± 2.3	120.4 ± 5.0	119.8 ± 5.2	122.3 ± 6.5
30 min after Air/NBO	121.9 ± 2.7	434.9 ± 10.8	120.4 ± 4.6	434.0 ± 13.9
3 hr after Air/NBO	121.5 ± 3.7	443.1 ± 13.2	119.9 ± 5.2	438.7 ± 20.8

pCO2 (mmHg)				
Before Air/NBO	45.3 ± 1.0	45.3 ± 1.6	45.9 ± 1.2	46.0 ± 1.4
30 min after Air/NBO	44.9 ± 0.8	44.6 ± 1.5	45.9 ± 1.5	45.7 ± 1.3
3 hr after Air/NBO	44.5 ± 1.2	45.3 ± 1.1	45.5 ± 1.3	45.1 ± 1.7

Values are mean ± SD. MABP indicates mean arterial blood pressure.

**Figure 2:**
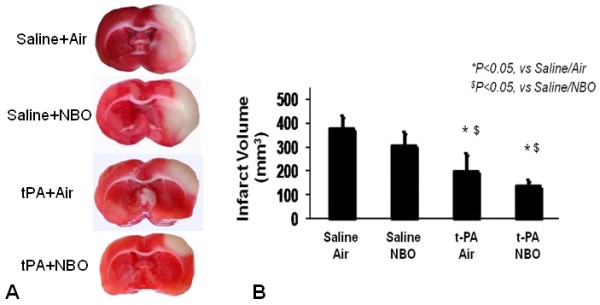
**Cerebral infarction volumes**. (a) Representative images of TTC staining are shown. (b) Effects of tPA and NBO on infarction volume at 24 hours. Intravenous tPA reduced infarct volumes but NBO did not alter this neuroprotective effect. Data expressed as mean ± SD; *^$ ^P < 0.05.

This embolic clot model yielded infarctions in the expected territory of the middle cerebral artery (Figure 2a). Treatment with NBO alone did not show a statistically significant decrease in infarction volume (309 ± 58 mm^3 ^in NBO animals, 379 ± 57 mm^3 ^in controls, Figure 2b). As expected, thrombolysis with tPA significantly decreased infarction volumes (201 ± 78 mm^3^), consistent with effective reperfusion. Combining NBO plus tPA did not interfere with this beneficial action of tPA. In fact, the average infarction volume of rats treated with NBO plus tPA (138 ± 30 mm^3^) was even smaller than that in tPA-alone rats (210 ± 78 mm^3^), although this difference was not statistically significant (p = 0.26). Both tPA-treated groups showed small cortical infarcts with subcortical tissue salvage (Figure 2A), suggesting thrombolysis-induced recanalization with distal clot migration or embolization, as expected for this experimental model[24].

**Figure 3:**
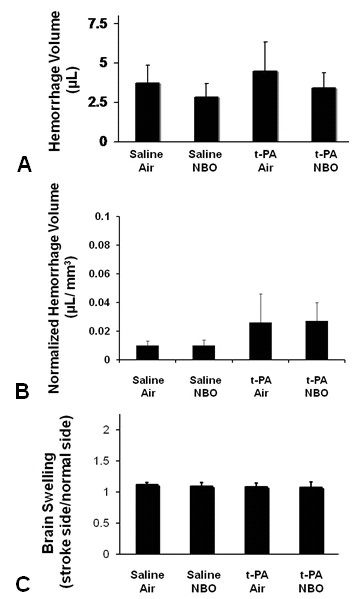
**Effects of tPA and NBO on brain hemorrhage and brain swelling**. Bar-graphs show the quantity of brain hemorrhage without (**a**) and with (**b**) correction for infarction volume, and (**c**) brain swelling at 24 hours. Intravenous tPA and NBO did not induce hemorrhagic conversion, and did not increase brain swelling.

In Spontaneously Hypertensive rats (SHRs), this embolic clot model demonstrates the presence of hemorrhagic conversion if thrombolytic reperfusion occurs late, at 2–3 hours or beyond. In this study, tPA was given relatively early, at 1 hour, which is the time point where reperfusion occurs consistently in our experience. Accordingly, there was no significant induction of hemorrhage. The calculated volumes of parenchymal blood were approximately 3–4 μL in all groups and neither NBO nor NBO plus tPA appeared to worsen hemorrhage (Figure 3a). After correcting for infarct volumes (Figure 3b), hemorrhage volume tended to be higher in both tPA treated groups as compared to saline controls (saline controls vs. tPA, p = 0.11, and vs. tPA/NBO, p = 0.09).

Besides hemorrhagic conversion, another potential complication of reperfusion injury is the induction of edema and brain swelling. In this study, about 10% of hemispheric swelling was observed in all animals. There were no detectable differences across groups, and NBO did not worsen brain swelling (Figure 3c). There was no significant difference in mortality rates between groups (p = 0.73), indicating that NBO did not affect mortality when administered alone or when combined with tPA (Table 2).

**Table 2:** Mortality Rates

Group	salile+Air	saline+NBO	tPA+Air	tPA+NBO
	n = 11	n = 9	n = 14	n = 10
				

dead	3	2	6	3
mortality rate	27%	22%	43%	30%

## Discussion

Over the past few years, multiple studies have documented NBO's neuroprotective effects in hyperacute focal ischemic stroke. Early NBO therapy salvages acutely ischemic brain tissue, reduces pathological brain infarct volumes, improves neurobehavioural scores, attenuates diffusion-weighted brain MRI abnormalities, and extends the reperfusion time window [3-16]. Pilot human studies have shown similarly promising results [17-19]. Other studies have shown that NBO does not induce post-ischemic brain hemorrhage, brain edema, blood-brain barrier damage, matrix metalloprotein levels, and various other markers of oxidative stress[6,9,12,15]; these results have helped to allay concerns about the potential risks of hyperoxia-induced oxygen free radical generation. Finally, several studies have shed light on mechanisms: NBO raises brain tissue oxygen levels, reduces peri-infarct depolarizations that contribute to infarct growth, improves MR-spectrophotometric parameters of ischemia e.g. brain lactate levels, and favorably alters hemodynamic parameters such as cerebral blood volume[8,11,13,14,18,19].

On basis of these data, and the inherent advantages of NBO as an acute stroke therapy such as its wide availability, ease of delivery in the field, and penetration across the blood brain barrier to reach target ischemic brain tissue, clinical trials of NBO have been initiated to determine NBO's safety and therapeutic potential in the acute stroke setting[17,18,25,26]. Combining NBO with tPA is a natural next step; however, before such trials can be initiated it is important to investigate in pre-clinical studies whether NBO influences the thrombolytic potency or the safety of tPA. In that regard, the results of the present study are significant. We found that the combination of NBO plus tPA did not significantly alter the rate or the degree of tPA-induced reperfusion as measured by LDF, and had no significant effect on brain swelling or mortality. Both tPA-treated groups showed significant reduction of infarct volumes as compared to saline controls, and the combination of NBO with tPA proved most effective. The benefit of combination therapy over tPA alone was marginal; however, this study was not powered to show benefit of combination therapy versus tPA. Both tPA groups showed small residual cortical infarcts as expected for this embolic clot-reperfusion stroke model[24]. NBO alone showed no significant benefit, presumably because of the small sample size, because it was delivered at a relatively delayed time frame (the therapeutic window in rodents is estimated at between 30 and 45 minutes[14]), and because LDF monitoring suggested non-reperfusion in the NBO-only group (prior work suggests that NBO' immediate tissue-salvaging effects cannot be sustained in the face of prolonged hypoperfusion[6,14]). As with previous studies, it is important to note that NBO also did not worsen infarct volume, showing lack of toxicity from mechanisms such as increased oxygen free radical generation.

These data complement the results of prior studies showing efficacy of NBO in mechanical models of reperfusion[4-6,8-15]. Because of the inherent differences between mechanical and thrombolytic approaches to arterial recanalization, here we used a clinically relevant stroke model of embolic MCA occlusion with tPA-induced thrombolysis within a narrow therapeutic time window. SHR rats were used since our main objective was to determine safety; the incidence of tPA induced hemorrhage is higher in SHR rats. Moreover, SHR rats develop more reproducible infarcts, which is an important consideration given that infarct size is more variable with the embolic clot model than with the filament model.

Our results are most applicable to the clinical setting where patients receive intravenous tPA and NBO *simultaneously *after hospital arrival. A previous rodent study modeling the clinical scenario where NBO is started in the field, followed by *delayed *tPA administration, has shown similarly promising results[7]. In that study, NBO was started at 30 minutes and tPA was administered at 3 hours after embolic MCA occlusion. Serial diffusion-perfusion magnetic resonance imaging (MRI) showed that the combination of NBO with tPA was effective (reduced the growth of diffusion-MRI lesions, smaller pathological infarct volumes) and also safe (no increase in the rate or the volume of brain hemorrhage). If confirmed in clinical trials, these data may result in the widespread use of NBO as an adjunctive stroke treatment strategy that extends the time window for intravenous tPA. NBO may eventually also have a role in extending the therapeutic time window for other promising treatments including intra-arterial thrombolytics and neuroprotective drugs, which to date have failed clinical trials due to factors such as delayed time to treatment, among others [27,28].

The strengths of our study include the blinded assessment of outcomes, careful monitoring of physiological parameters and cerebral perfusion, statistical rigor, and the clinically relevant study design. These factors are often overlooked in pre-clinical studies [29,30]. We acknowledge several shortcomings, for example the absence of neurobehavioural scores and investigation of mechanisms. These issues have been addressed in prior studies; our objective was to investigate the interaction between NBO and intravenous tPA. Further studies are warranted to investigate issues such as the effect of varying durations, timings, and concentrations of inhaled oxygen, and intravenous tPA's therapeutic time window with NBO pre-treatment and co-treatment. Nevertheless, we hope that our results will lend confidence to starting clinical investigations of NBO with tPA.

## Conclusion

These experimental results suggest that NBO can be safely co-administered with intravenous tPA to treat acute ischemic stroke. The efficacy of tPA thrombolysis is not affected by NBO, and there is no induction of brain hemorrhage or brain edema.

## Methods

### Animal Model

All experiments were performed under Protocol No. 2006N000138 approved by the Massachusetts General Hospital Subcommittee on Research Animal Care, and in accordance with the internationally recognized National Institutes of Health (NIH) Guide for the Care and Use of Laboratory Animals. Spontaneously hypertensive male rats (Charles River Laboratories, Wilmington, MA) were anesthetized with isoflurane (5% induction, 1–1.2% maintenance) in a 30% oxygen and 70% nitrous oxide mix. Temperature was maintained at 37 ± 0.5°C with a heating pad. Right femoral arteries were cannulated to monitor pressure, pH and gases. The embolic stroke model was adapted from Zhang, et al.[31] Briefly, 3 cm of homologous clots were injected via a modified PE-50 catheter to occlude the middle cerebral artery (MCA). After embolization, animals were randomly allocated to one of four treatment groups: intravenous saline injected at 1 hour after ischemia; intravenous tPA injected at 1 hour after ischemia; saline injected at 1 hour after ischemia plus NBO; tPA injected at 1 hour after ischemia plus NBO.

Consistent with STAIR recommendations[29], the experiment was performed in blinded fashion, as follows. Randomization was performed after embolization in order to confirm successful clot placement and drop in laser Doppler flowmetry (LDF) values. The investigator performing the surgery (YM) left the room after clot placement. A second 'unblinded' investigator (NF or KA) performed the randomization, administered the intravenous solution, manipulated gas flow rates, obtained arterial blood gases, and adjusted the anesthesia or flow of gas if warranted. The room air and oxygen flowmeters were covered in order to protect the blind. The blinded investigator (YM) then returned to complete the surgical procedure and monitor HR, BP, and LDF during the period of anesthesia, but did not view potentially unblinding information such as flow of gas or arterial blood gas results. A separate set of blinded investigators (JL, YE) and the blinded surgeon (YM) assessed outcomes at 24 hours. If the animal died before the 24-hour time point, the unblinded investigators (NF, KA) were informed and the number of animals in that group adjusted accordingly.

Intravenous tPA (Genentech, San Francisco, CA) was administered at 10 mg/kg with a 10% bolus and 90% continuous infusion over 30 minutes. LDF probes placed 2 mm posterior and 5 mm lateral to the bregma were used to monitor cerebral perfusion. Only rats that showed sustained ischemia to less than 20% of pre-ischemic baselines until the time of injection were included. Animals with LDF value changes suggesting spontaneous recanalization before saline or tPA injection were excluded. All gases were delivered via a simple face mask to free-breathing animals. NBO was achieved by stopping nitrous oxide and increasing the concentration of oxygen to 100%. NBO was started simultaneously with saline or tPA injection (at 1 hour) and continued for a duration of 3 hours. At 4 hours after MCA occlusion (3 hours after saline or tPA injection), anesthesia was discontinued and rats were returned to their cages.

### Measurement of Infarction, Brain swelling and Intracranial Hemorrhage

All rats were sacrificed at 24 hours after ischemia under deep pentobarbital anesthesia, brains were transcardially perfused with saline, and seven coronal sections (2 mm thick) were stained with 2,3,5-triphenyltetrazolium chloride (TTC; Sigma, St. Louis, MO) to quantify infarct volumes as described previously [14,15]. The degree of brain swelling was estimated as the volumetric ratio of the ischemic side divided by the contra-lateral side. Cerebral hemorrhage was measured using a spectrophotometric assay to quantify hemoglobin in perfused brain[15]. We have previously shown that hemoglobin measurements can be simultaneously performed on TTC-stained sections[32].

### Statistical Methods

Power and sample size calculations, based on the expected size and standard deviation for infarct volumes in prior experiments and the anticipated effect size for tPA versus saline controls, suggested that 7 to 8 animals per group would be required to achieve statistical significance. Accordingly, the surgical experiments were continued until we reached at least 7 rats per group that survived until 24 hours and were not initially excluded due to spontaneous recanalization. Continuous variables (physiological parameters, LDF values, and infarction, brain edema, and hemorrhage volumes) were analyzed with ANOVA followed by Tukey-Kramer tests. Mortality rates were compared using the Chi-square test. Differences with *P *< 0.05 were considered statistically significant. Data are expressed as mean ± SD.

## Abbreviations

tPA: tissue plasminogen activator; FDA: Food and Drug Administration; NBO: normobaric oxygen therapy; NIH: National Institutes of Health; MCA: middle cerebral artery; STAIR: stroke therapy academic industry roundtable; LDF: laser Doppler flowmetry; TTC: 2,3,5-triphenyltetrazolium chloride; SD: standard deviation; SHR: spontaneously hypertensive rats; MRI: magnetic resonance imaging.

## Authors' contributions

NF, YM, KA, YE and JL carried out the surgical experiments and assessed outcomes in blinded fashion as described in the Methods section. OW participated in study design and coordination, and helped to draft the manuscript. ABS and EHL designed the study, obtained funding, supervised the experiments, interpreted the data, and drafted the manuscript. All authors read and approved the final manuscript.
